# Jugular vs femoral vein for central venous catheterization in pediatric cardiac surgery (PRECiSE): study protocol for a randomized controlled trial

**DOI:** 10.1186/s13063-018-2717-1

**Published:** 2018-06-25

**Authors:** Simona Silvetti, Tommaso Aloisio, Anna Cazzaniga, Marco Ranucci

**Affiliations:** Department of Cardiovascular Anesthesia and Intensive Care, IRCCS Policlinico San Donato, Via Morandi 30, 20097 San Donato Milanese, Milan, Italy

**Keywords:** Central line–associated bloodstream infection (CLABSI), Catheter-related bloodstream infection (CRBSI), Congenital heart disease, Infection, Newborn

## Abstract

**Background:**

Placement of central venous catheters (CVCs) is essential and routine practice in the management of children with congenital heart disease. The purpose of the present protocol is to evaluate the risk for infectious complications in terms of catheter colonization, catheter line–associated bloodstream infections, and catheter-related bloodstream infections (CRBSIs), and the mechanical complications from different central venous access sites in infants and newborns undergoing cardiac surgery.

**Methods:**

One hundred sixty patients under 1 year of age and scheduled for cardiac surgery will be included in this randomized controlled trial (RCT); patients will be randomly allocated to the jugular or femoral vein arms. CVC insertion will be performed by one of three selected expert operators.

**Discussion:**

The choice of the insertion site for central venous catheterization can influence the incidence and type of infectious complications in adults but this is not unanimously evidenced in the pediatric setting. The experimental hypothesis of this RCT is that the jugular insertion site is less likely to induce catheter colonization and CRBSI than the femoral site.

**Trial registration:**

ClinicalTrials.gov Identifier: NCT03282292. Registered on 12 September 2017.

**Electronic supplementary material:**

The online version of this article (10.1186/s13063-018-2717-1) contains supplementary material, which is available to authorized users.

## Background

Central venous catheter (CVC) placement is a routine technique in the management of children undergoing cardiac surgery. This procedure may be difficult and challenging in small-sized patients and carries an associated morbidity and mortality [1, 2]. For this reason, a number of studies in different settings and in different pediatric populations were performed to investigate the complications related to CVC placement [3–6]. The occurrence of complications, particularly infections, and success rate will depend on factors which include the size and condition of the child, operator experience, and the site of cannulation.

Children with congenital heart disease may have a different risk profile in terms of infection: children undergoing cardiac surgery are often newborn or small-sized, mainly undergo major surgical procedures, and suffer from the inflammatory and immunosuppressive effects of cardiopulmonary bypass; moreover, the perioperative period is frequently characterized by the use of multiple invasive devices.

Although in the multidisciplinary adult setting the femoral vein (FV) was associated with a higher risk of infections [7] and excluded as the first choice from the existing guidelines [8], this result was not confirmed in pediatric studies [6] and not defined in the vascular access guidelines [8].

Also, in terms of mechanical complication, there is univocal information. Recently, Karapinar and Cura [9] concluded that, for central venous catheterization, it is better to initially choose the femoral or internal jugular vein (JV) instead of the subclavian vein because of a higher success rate without serious insertion-related complications. In contrast, in a recent randomized controlled trial, Camkiran Firat and colleagues [10] found that central venous catheterization through the internal JV and subclavian vein was not significantly different in terms of mechanical complications.

The primary hypothesis of the present study is that the FV access for CVC carries a higher risk for catheter colonization and consequently for catheter-related bloodstream infection (CRBSI) than the internal JV access in newborns and infants undergoing cardiac surgery. In this study, we would also value the impact of insertion site (jugular or femoral) on the development of mechanical complications**.**

## Methods

### Study design

This study is a prospective randomized controlled trial with two arms of treatment and follow-up until hospital discharge. The study was approved by the local ethics committee of IRCCS San Raffaele Hospital (version 1.1 19/06/2017, amended into version 1.2 20/10/2017). The study was registered at ClinicalTrials.gov (NCT03282292). The study was funded by research funds from IRCCS Policlinico San Donato, a clinical research hospital partially funded by the Italian Ministry of Health. The study will be conducted in IRCCS Policlinico San Donato, a tertiary care cardiac surgery hospital.

The integrity and quality of data, and the assessment of adverse events, will be reviewed by an internal data monitoring committee.

**Fig. 1:**
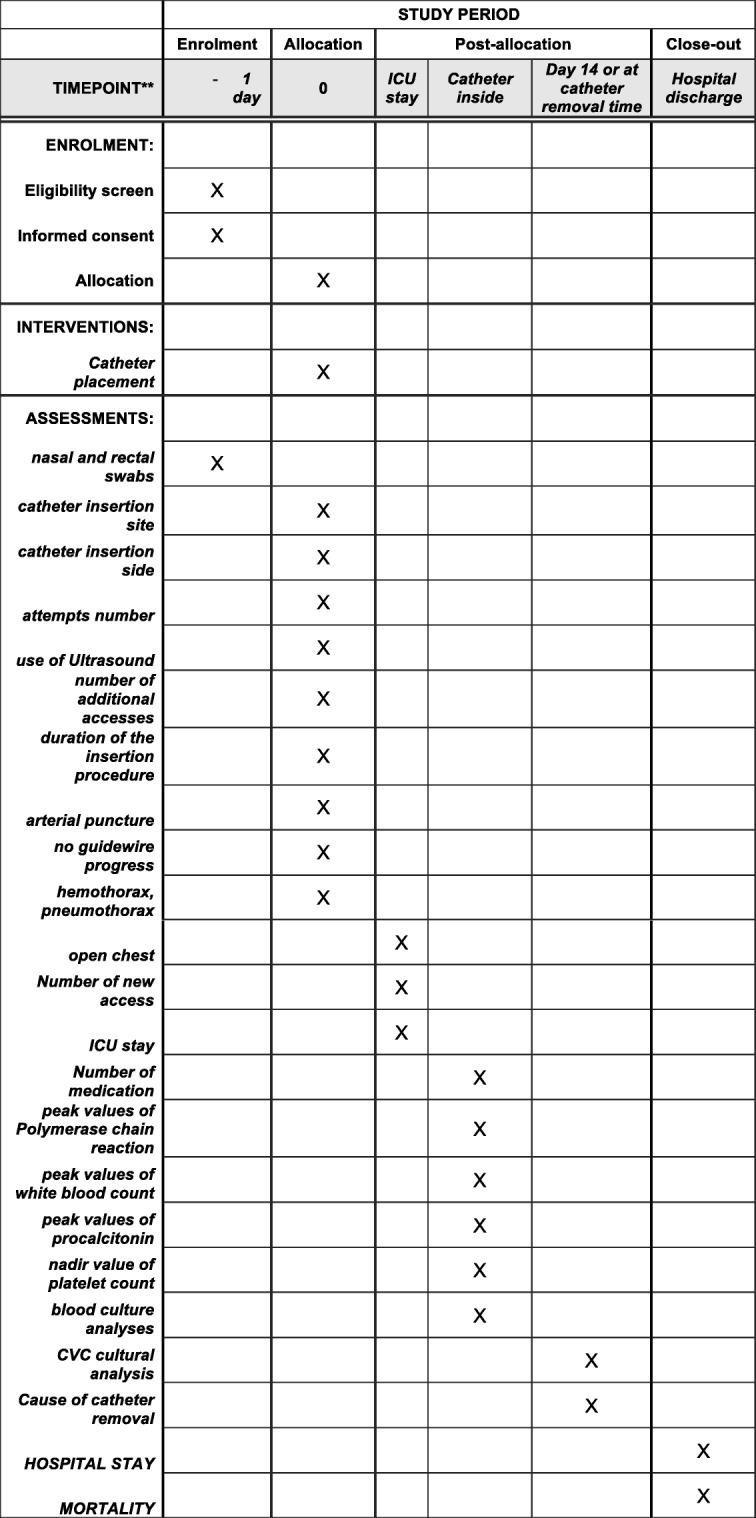
Standard Protocol Items: Recommendation for Interventional Trials (SPIRIT) schedule of enrollment, interventions and assessments

Figure 1 shows the Standard Protocol Items: Recommendation for Interventional Trials (SPIRIT) schedule of enrollment, interventions, and assessments. The SPIRIT Checklist is presented in Additional file 1.

### Study population

Inclusion and exclusion criteria are presented in Table 1. Recruitment started in September 2017 and is likely to be completed in September 2019.

**Table 1:** Elegibility criteria

Inclusion criteria	Planned cardiac surgery Age < 1 year Eligibility for both insertion sites (jugular and femoral) for central venous catheter (CVC) Availability of at least one of the three chosen expert operators
Exclusion criteria	Emergency surgery Known vascular anatomic anomalies Previous cardiac surgery in the last 6 months No expert operator available Intensive care unit before surgery CVC inside at the time of randomization Signs of sepsis
Withdraw criterion (only for the first endpoint)	Impossibility to placement catheter in the selected site.

### Recruitment and study flow

The patients are screened at the hospital admission by one cardiac anesthesiologist (SS). If the patient is eligible according to the criteria of Table 1, the parents are addressed, the study protocol is explained by the same anesthesiologist, and written informed consent to participate in the study is collected. Anonymous data analysis is guaranteed by blinding data for identification. There are no limits about other care and interventions that are permitted or prohibited during the trial.

### Randomization

A randomization code was generated with a computerized system producing sealed envelopes containing the indication for the allocation to the JV or FV arms. The envelopes were prepared by a study coordinator not directly involved in the trial-related procedures following the opening of the envelopes. The randomization list was created in blocks of 20 subjects. The study was open-label.

### Sample size

The power analysis was conducted on the basis of the endpoint of catheter colonization. Previous observational data on catheter line–associated bloodstream infections (CLABSIs) in a non-surgical neonatal intensive care showed that the femoral line was associated with a CLABSI rate of 10.2% but that the jugular site had a CLABSI rate of 2.7% [11]. We assumed a 2:1 ratio between catheter colonization rate and CLABSI rate and therefore hypothesized colonization rates of 20.4% for the femoral line and 5.4% for the jugular line. With an alpha error value of 5% and a power of 80%, 77 patients are required for each group. Considering a dropout rate of 4%, we increased this number to 80 subjects for each group.

### Study intervention

#### Intraoperative management

The envelopes containing the randomized allocation, JV or FV arms for the central venous catheterization, were opened immediately before surgery by one of three chosen expert operators who will perform the procedure (MR, AC, and TA).

Each patient receives our standard perioperative antimicrobial prophylaxis with intravenous cefazolin (30 mg/kg 60 min before the procedure) or cefazolin plus vancomycin 15 mg/kg when methicillin-resistant *Staphylococcus aureus* (MRSA) was found in the preoperative nasal swab.

Anesthesia is carried out in accordance with our institutional practice: induction with intravenous midazolam or propofol, followed by a continuous infusion of the same agent plus opioids (fentanyl or sufentanil according to the age). Additional inhalational agents (sevoflurane) can be used as appropriate. Neuromuscular blockade is achieved with cisatracurium or rocuronium. All patients are endotracheally intubated and mechanically ventilated. Standard monitoring is used and this includes a radial (first choice) or femoral (second choice) artery catheter for measurement of systemic arterial blood pressure and intermittent blood sampling; if the randomly assigned patient is in the FV arm, a different side is chosen for artery and vein cannulation: an additional single lumen venous access in the peripheral or central vein to allow a high-volume infusion and esophageal and rectal temperature probes.

#### Catheter placement

All procedures are conducted in the operating room by one of three chosen expert operators (MR, AC, and TA). After the random allocation to the JV or FV arm for CVC, the chosen site (femoral or jugular) is prepared by using ChloraPrep® (Carefusion UK, San Diego, CA, USA), and the procedure is conducted with maximal sterile barrier precautions [8]. The side (right or left) is chosen by the anesthesiologist in charge. In both JV and FV, a PediaSat® catheter (Edwards Lifesciences, Irvine, CA, USA) double lumen (length of 5 or 8 cm) is placed. This kind of CVC was chosen to be in keeping with our standard monitoring procedure, which includes continuous central venous oxygen saturation (ScvO_2_) monitoring in the operating room and intensive care unit (ICU) [12].

**Fig. 2:**
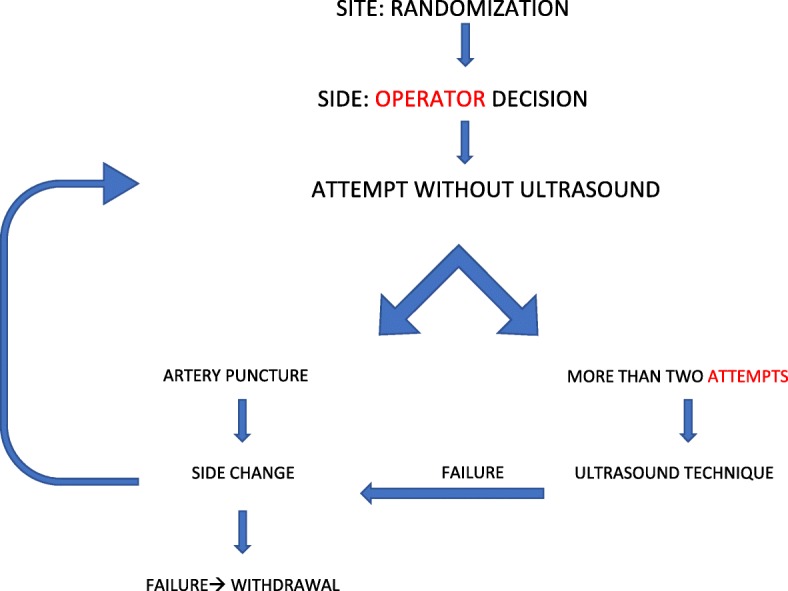
Central venous catheter placement procedure

The CVC placement procedure is described in Fig. 2: a landmark approach is accepted for two attempts, the third attempt in the same side must be performed by ultrasound (US)-guided techniques, and VividE (GE, Fairfield, CT, USA) and a 6- to 12-MHz linear-array US probe are used. If an accidental arterial puncture occurs or if the vein visualized by US was considered inadequate in size by the operator, the insertion side is changed. If the catheter placement fails in the other side also (after two landmark attempts and the third attempt with US), the patient will be withdrawn from the study.

All CVCs are fixed to the skin by suture stitches.

All medications are performed by nurses at the end of surgery in the operating room: cleaning of the site is performed with clorexidine 2%, a BIOPATCH® (Ethicon US, LLC, Bridgewater, NJ, USA) is positioned, and a sterile transparent polyurethane adhesive wound dressing is used to cover the catheter exit site.

#### Catheter and patient management

**Fig. 3:**
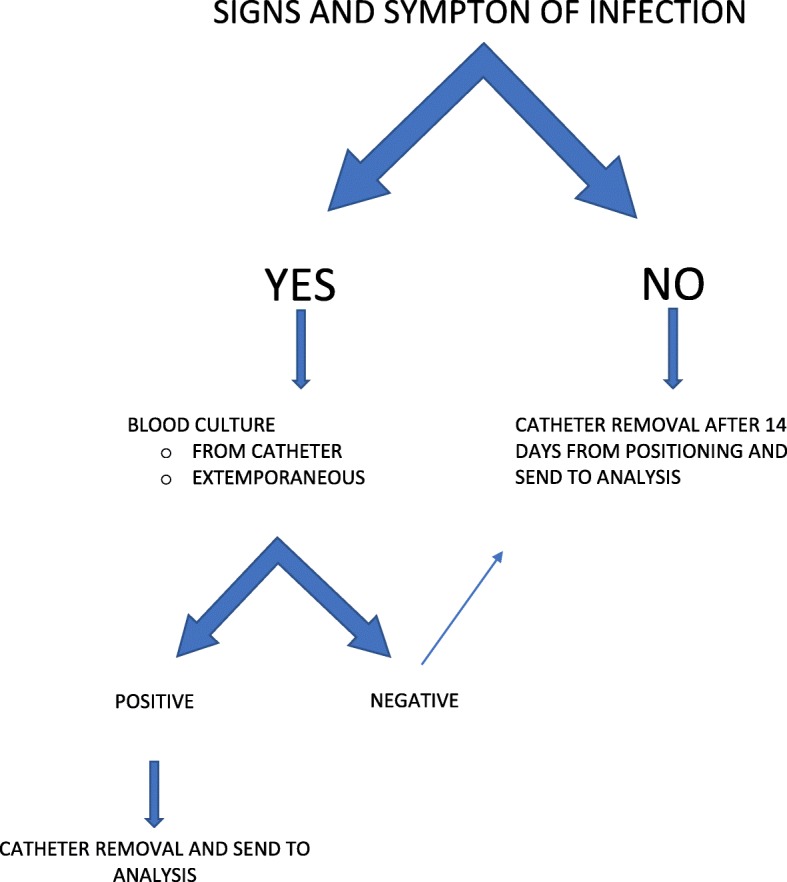
Central venous catheter management

After open heart surgery, all patients are admitted to the pediatric ICU for the usual care. The CVC is managed as follows (Fig. 3): the CVCs are removed when useless but not later than the 14th day from the positioning in the operating room. Once removed, all CVCs are sent for a culture analysis. If signs and symptoms of infection are found, the blood culture is performed from the CVC and whenever possible from a new venipuncture. Sometimes it could not be done: when CLABSI is suspected (8 days after procedure [13]), the babies are usually awake; this makes a new venipuncture cruel and is not easy without sedation. Unfortunately, without a blood culture from a new venipuncture, we cannot discriminate the infection’s origin.

If the blood culture from CVC is positive, the CVC is removed and sent for culture analysis.

All catheter medical treatments are performed by nurses every 7 days or if visibly dirty: after cleaning of the site with clorexidine 2%, a BIOPATCH® (Ethicon US, LLC) is positioned and a sterile transparent polyurethane adhesive wound dressing is used to cover the catheter exit site.

The postoperative antimicrobial prophylaxis consists of cefazolin 100 mg/kg fractionated in three daily doses and is continued no longer than 48 h. Vancomycin prophylaxis is limited to the preoperative dose. In open-chest patients, the prophylaxis is based on vancomycin 15 mg/kg four times daily plus amikacin 15 mg/kg one time daily plus ceftazidime 30 mg/kg three times daily until 1 day after sternal closure.

### Measurements and definitions

Data collection is based on our institutional database, which includes all the demographics, preoperative factors, procedural details, intraoperative data, and outcome measurements.

For the purposes of the present study, the following parameters are measured:

- Preoperative nasal and rectal swabs (when performed).
- Procedural data: catheter insertion site and side; number of attempts; use of US; number of additional accesses; and duration of the insertion procedure.
- Procedural complications defined as arterial puncture, no guidewire progress; hemothorax, pneumothorax.
- Intraoperative data: open chest at transfer to the ICU.
- Postoperative data: peak values of polymerase chain reaction, white blood count, procalcitonin, and nadir value of platelet count, collected as a sign of infection; time to peak from CVC insertion through removal; results of the blood culture analyses performed if signs and symptoms of infections are found; CVC cultural analysis performed for all catheters at removal time. Cause of catheter removal (accidental, end of use, or CLABSI). The number of new accesses positioned in the ICU is also recorded.
- No follow-up will be performed after catheter removal.

Definitions:

- Catheter colonization: bacterial growth identified at the CVC culture at removal time.
- CLABSI: a laboratory-confirmed bloodstream infection when central line was in place for more than 48 h.
- CRSBI is the identification of the same bacterial in blood culture and in the catheter tip after the catheter removal

Measures for data quality assurance, patient retention, and protection.

- General outcome data are retrieved by our institutional database managed by trained personnel. Data specific to this study (i.e., blood culture and CVC culture) are the responsibility of trained personnel of our clinical laboratory, and their interpretation will be supported by consultation with our local specialist in infectious disease.
- No specific plans for patient retention, avoidance of spontaneous withdrawal, or loss at follow-up were implemented since the observation time is limited to the hospital stay.
- Given that both the study arms receive standard procedures, no specific insurance was requested by our ethics committee. The patients are protected for potential damages by our Institutional Insurance as requested by Italian Law.

### Study endpoints

The primary endpoint is the catheter colonization, CLABSI and CRBSI rate.

Secondary endpoints are procedural complications rate and procedural difficulties rate.

### Statistics

Homogeneity of the two study arms will be tested with parametric (Student *t* test) and non-parametric (Mann-Whitney *U* test) tests depending on the normality of distribution of the continuous variables and with a Pearson’s chi-squared or a Fisher exact test for categorical variables.

Colonization and CLABSI and CRBSI rate will be investigated as binary variables and expressed as number and percentage. The difference in colonization and CLABSI and CRBSI rate between the two study arms will be tested at univariate analysis by using a Pearson’s chi-squared or a Fisher exact test as appropriate, producing relative risks and 95% confidence intervals.

In case of inhomogeneity of the study arms, the factor(s) being significantly different between arms will be used as potential confounders in a multivariable logistic regression model, producing odds ratio and 95% confidence interval.

Time-dependent colonization and CLABSI occurrence will be estimated by using a Kaplan-Meier analysis, and the two arms will be compared by using a log-rank test. In case of inhomogeneity between the two arms, a multivariable Cox regression analysis will be applied.

Secondary endpoints will be treated as binary variables according to the same statistical approach. For all the statistical tests, a *P* value of less than 0.05 will be considered significant. Computerized statistical programs will be used for the analysis.

## Discussion

It is currently clear that in adult patients the FV is the last choice for central venous catheterization because of an increased infection risk [7, 8]. In contrast, at the moment, the choice of the anatomic site in the pediatric patient population depends mainly on the operator’s preference and local policies. Few studies have been published about this topic [3, 10, 13], and none assessed the differences between FV and JV in a small pediatric cardiac population. Owing to the frequent utilization of CVC and given some relevant specific postoperative conditions for these kinds of patients (for example, the need for the open chest at the end of surgery), it has become essential to identify which kind of site is the safest in terms of infectious and mechanical risk.

The primary endpoint of our study is to quantify the catheter colonization and CLABSI and CRBSI rate according to insertion site. At the end of this study, we could validate the best anatomic site in pediatric cardiac surgery patients in terms of infectious risk. In the present era of nosocomial infection pandemia, this information could represent a useful insight to be included in the institutional protocols for the prevention of CRBSI.

Additional data may derive from the secondary endpoints and basically from the potential differences in procedural complications. The combination of infectious and insertion risks could be used on a case-by-case basis to facilitate the decision-making process on the insertion site. A comprehensive decision should include an analysis of the general infectious risk (including the weight of the surgical procedure and the expected CVC permanence), of the potential insertion-related complications, and of the potentially different degree of difficulty in the insertion.

In this respect, there are certainly some limitations in our protocol. The main one is the obvious fact that a CVC insertion in a small infant is a process which involves dexterity and expertise. Moreover, it could be that the operator has different levels of confidence with the different sites of insertion. Owing to this inevitable bias, our results may not necessarily be generalizable, especially with respect to the dexterity-related endpoints of insertion success, time, and complications.

## Trial status

Study start: September 12, 2017.

Primary completion: September 12, 2019.

## Additional file

Additional file 1: Standard Protocol Items: Recommendation for Interventional Trials (SPIRIT) 2013 Checklist. (PDF 69 kb)

## **Abbreviations**

CLABSI: Catheter line–associated bloodstream infection
CRBSI: Catheter-related bloodstream infection
CVC: Central venous catheter
FV: Femoral vein
ICU: Intensive care unit
JV: Jugular vein
SPIRIT: Standard Protocol Items: Recommendation for Interventional Trials
US: Ultrasound

## References

[1] Stenzel JP, Green TP, Fuhrman BP, Carlson PE, Marchessault RP (1989). Percutaneous central venous catheterization in a pediatric intensive care unit: a survival analysis of complications. Crit Care Med..

[2] Odetola FO, Moler FW, Dechert RE, VanDerElzen K, Chenoweth C (2003). Nosocomial catheter-related bloodstream infections in a pediatric intensive care unit: risk and rates associated with various intravascular technologies. Pediatr Crit Care Med..

[3] Costello JM, Graham DA, Morrow DF, Potter-Bynoe G, Sandora TJ, Laussen PC (2009). Risk factors for central line-associated bloodstream infection in a pediatric cardiac intensive care unit. Pediatr Crit Care Med..

[4] de Jonge RC, Polderman KH, Gemke RJ (2005). Central venous catheter use in the pediatric patient: mechanical and infectious complications. Pediatr Crit Care Med..

[5] Miguelena D, Pardo R, Morón-Duarte LS (2013). Central venous catheter-related complications in critically ill children. Rev Salud Publica..

[6] Casado-Flores J, Barja J, Martino R, Serrano A, Valdivielso A (2001). Complications of central venous catheterization in critically ill children. Pediatr Crit Care Med..

[7] American Society of Anesthesiologists Task Force on Central Venous Access (2012). Practice guidelines for central venous access: a report by the American Society of Anesthesiologists Task Force on Central Venous Access. Anesthesiology..

[8] Centers for Disease Control and Prevention. Guidelines for the Prevention of Intravascular Catheter-Related Infections. 2011 https://www.cdc.gov/infectioncontrol/guidelines/BSI/index.html. Accessed 15 Nov 2017.

[9] Karapinar B, Cura A (2007). Complications of central venous catheterization in critically ill children. Pediatr Int..

[10] Camkiran Firat A, Zeyneloglu P, Ozkan M, Pirat A (2016). A randomized controlled comparison of the internal jugular vein and the subclavian vein as access sites for central venous catheterization in pediatric cardiac surgery. Pediatr Crit Care Med..

[11] Freeman JJ, Gadepalli SK, Siddiqui SM, Jarboe MD, Hirschl RB (2015). Improving central line infection rates in the neonatal intensive care unit: effect of hospital location, site of insertion, and implementation of catheter-associated bloodstream infection protocols. J Pediatr Surg..

[12] Ranucci M, Isgrò G, De La Torre T, Romitti F, De Benedetti D, Carlucci C, Kandil H, Ballotta A (2008). Continuous monitoring of central venous oxygen saturation (Pediasat) in pediatric patients undergoing cardiac surgery: a validation study of a new technology. J Cardiothorac Vasc Anesth..

[13] Aiyagari R, Song JY, Donohue JE, Yu S, Gaies MG (2012). Central venous catheter-associated complications in infants with single ventricle: comparison of umbilical and femoral venous access routes. Pediatr Crit Care Med..

